# Dendrimer-Based Nanomedicine (Paramagnetic Nanoparticle, Nanocombretastatin, Nanocurcumin) for Glioblastoma Multiforme Imaging and Therapy

**DOI:** 10.31031/nacs.2021.06.000639

**Published:** 2021-10-20

**Authors:** Stephen L Brown, James M Snyder, Meser M Ali

**Affiliations:** Department of Neurosurgery, USA

**Keywords:** Dendrimer, Nanomedicine, Glioma, Cancer imaging and therapy

## Abstract

In brain tumors, delivering nanoparticles across the blood-brain tumor barrier presents a major challenge. Dual mode magnetic resonance imaging and fluorescent imaging probes have been developed where relaxation based Gd-DOTA or ParaCEST agents and a Near-Infrared (NIR) fluorescent dye, DL680 were conjugated on the surface of dendrimer. The *in vivo* and *ex vivo* imaging of the dual-modality contrast agent showed excellent potential utility for identifying the location of glioma tumors. Systemic delivery of the subsequent nano-sized agent demonstrated glioma-specific accumulation, probably due to the enhanced permeability and retention effect. The biodistribution studies revealed the G5 agents have accumulated in the glioma tumor and the liver while a G3 agent only accumulated in the brain tumor but not in the liver or kidney. Hydrophobic drug molecules like Combrestatin A4 (CA4) or curcumin have also been conjugated with dendrimers that provided high aqueous solubility with improved therapeutic effect.

## Introduction

Glioblastoma Multiforme (GBM, World Health Organization/ WHO grade IV) is the most common malignant primary brain tumor in adults [1]. GBM is the most common primary tumor of the CNS, and accounts for 12–15% of all intracranial tumors and 50–60% of gliomas [2]. GBM is characterized by molecular heterogeneity and the poorest prognosis [2]. Despite the advances made in therapeutic options for GBM, its prognosis remains poor with an overall survival rate (<2 years) that has been stagnant for three decades at approximately 20%. It is one of the unfortunate cancers where no predominant genetic alteration has been identified that could be targeted, resulting in limited therapeutic options. The multimodal treatment of GBM includes maximal surgical resection followed by Radiotherapy (RT) Plus Temozolomide (TMZ) chemotherapy, which may increase median survival to 12–15 months, although the disease typically progresses within 6–9 months, and 2-year survival is less than 25% [3,4].

Invasive biopsy is routinely utilized to assess histological type, classification, grade and potential aggressiveness of brain cancer and also for determination of the type of drug regimen employed for treatment [5,6]. Imaging techniques include Computed Tomography (CT), Positron Emission Tomography (PET), ultrasound and, most importantly, Magnetic Resonance Imaging (MRI) [7–11]. For some brain tumors the delineation of the actual tumor volume is difficult because peritumoral edema does not readily allow precise discrimination of tumor margins [12]. The use of contrast agent helps overcome this deficiency and allows estimates of tumor margins [13–15]. However, such tumor enhancement using contrast agents is possible only in patients with a compromised Blood-Brain Barrier (BBB) [12]. One approach that utilizes the unique structural features of many solid tumors (hypervasculature, defective vascular architecture, and impaired lymphatic drainage) [16,17] may lead to relatively selective extravasation and retention of long circulating nanocarriers. This phenomenon (“passive targeting”) is essentially the working principle of most clinically viable targeting strategies based on nanocarriers. This is the Enhanced Permeation and Retention (EPR) effect and has been described for nanoparticulate systems including liposomes, dendrimers, micelles, and polymers [18–20].

Brain tumors demonstrate high levels of angiogenic activity resulting in formation of torturous and abnormally dilated vessels with leaky inter-endothelial gaps and fenestration [21–23]. This hyper-permeable vasculature allows nanoparticles to extravasate and be retained in tumor interstitium following systemic administration [24]. Yet, effective transvascular delivery of nanoparticles across the Blood-Brain Tumor Barrier (BBTB) of malignant gliomas remains a challenge. We and others recently developed dendrimer-based paramagnetic nanoparticles that were found to preferentially accumulate in an orthotopic preclinical glioma model with a compromised BBTB [23,25,26]. These dendrimer-based polymers have the advantage of small particle size [25,26] (~7–12nm) and therefore the potential to improve tumor penetration. Drug molecules can also be conjugated on the surface of dendrimer structure and have the potential for lower systemic toxicity. Hydrophobic anti-cancer drugs like combretastatin a4 (CA4) and Curcumin (Cur) have been conjugated on the surface of a Generation 3 (G3) dendrimer that provide high water solubility and bioavailability with improved therapeutic effects [27,28]. Here we review the feasibility of dendrimer-based nanomedicine for glioma imaging and therapy.

### Dendrimer-Based Dual Mode Imaging Agent

Poly (Amidoamine) (PAMAM) dendrimers have been widely used for biomedical applications. This class of polymers has several favorable properties including a well-defined chemical structure, globular shape, low polydispersity index, biocompatibility, and controlled terminal functional groups. Modification of the PAMAM dendrimer surface functional groups with targeting compounds, fluorescent groups, and drugs have produced promising imaging and therapeutic agents [29]. The pharmacokinetics of the dendrimers have been alerted by conjugating non-scaffold polymers such as Polyethylene Glycol (PEG). Oxygen rich PEGs interact with water through hydrogen bonding that minimizes nonspecific interaction with proteins and other biological molecules in the circulation. Moreover, the introduction of PEGs on the surface of dendrimers can reduce the access of the enzymes at the close proximity of the dendrimer conjugates that provides the stability of the loaded drugs/genes from *in vivo* biodegradation [30].

Intravenous administration of a generation five (G5) PAMAM dendrimer labelled with tritium showed renal-based excretion [31]. Malik et al. [32] reported that I125-labeled PAMAM dendrimer demonstrated 60% of dendrimer accumulation in the liver and only 1% of the injected dose remained in the blood circulation one hour after administration [32]. In contrast, introduction of PEGs on the amines surface of PAMAM dendrimer exhibited longer blood half-life [33]. However, dendrimer-based drug delivery cannot be expected to be equally effective across tumor types, sizes, locations, stages and grades. Dendrimers were decorated with relaxation-based MRI contrast agents [24,25,34,35]. Paramagnetic Chemical Exchange Saturation Transfer (PARACEST) agents23 and Biosensor Imaging of Redundant Deviation in Shifts (BIRDS) agents [36–38].

**Figure 1:**
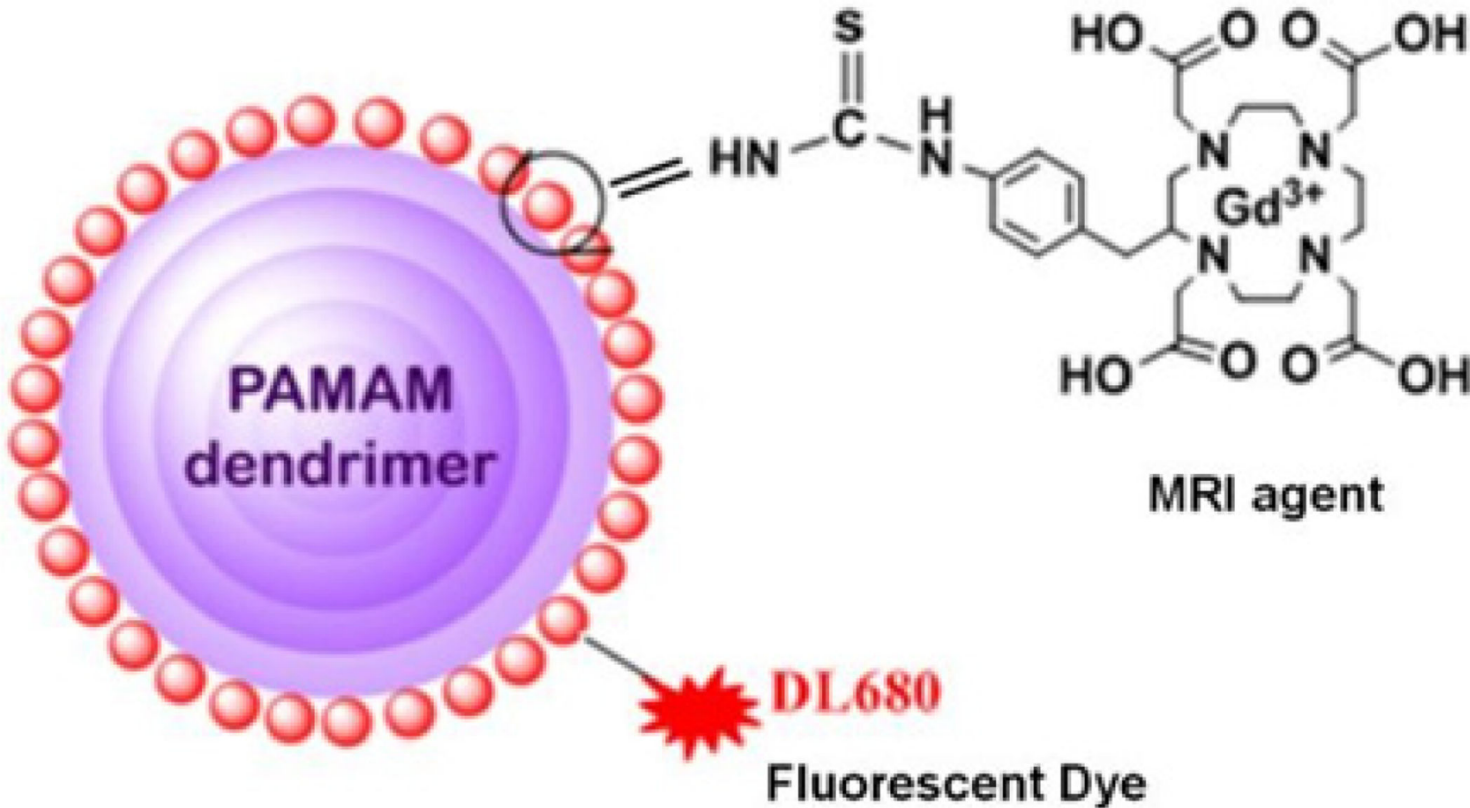
Schematic view of dual-mode dendritic conjugate [(Gd-DOTA)54-G5- DL680]. MRI contrast agent, Gd-DOTA is conjugated with a G5 PAMAM dendrimer. A fluorescent dye, DyLight (DL680), is also conjugated with the same dendrimer.

In a recent study a series of PAMAM dendrimer-based Gn-Gd-DTPA (G1 to G8) were synthesized, and the pharmacokinetics of the synthesized agents were studied in the BBTB of glioma tumor-bearing rats [24,34,35]. The properties of dendrimer-based Gd-DTPA agents *in vivo* depend on the size, core and exterior surface charge [39,40] and the porosity and pore size of tumor vessels vary with the type and status of the tumor. It was demonstrated that gadolinium chelated dendrimer nanoparticles with core sizes of <12nm permeated the BBTB, whereas larger nanoparticles were hindered [41]; thus, the upper limit of pore size in the BBTB of malignant brain tumors is approximately 12nm [24,42,43]. Spherical dendrimer-based paramagnetic nanoparticles ranging between 4 to 10nm in diameter maintain peak blood concentrations for several hours [24,41,44]. Gadolinium-based contrast agents have been widely used as contrast media for MRI. But free Gd3+ ions are toxic to biological systems and a suitable ligand or chelate must bind the lanthanide to form a bio-unavailable and nontoxic complex. In 2006, concern associated with the use of Gd3+ agents were reported due to an apparent link to a disabling condition known as nephrogenic systemic fibrosis (NSF) [45–47]. Two clinically approved agents - omniscan and magnevist - have been associated with NSF [47]. These are all linear Gd3+ chelates based on the structure of Diethylenetriamine Penta-Acetic Acid (DTPA) which is a less thermodynamically stable linear acyclic ligand than macrocyclic chelators. Beyond NSF, safety concerns regarding the retention of Gd3+ in the Central Nervous System (CNS) have intensified after cases of prolonged signal enhancement in the brain were reported, particularly in patients experiencing repeated contrast media administration [48,49]. Therefore, much attention has been paid to the development of thermodynamically stable macrocyclic Gd-DOTA based chelates. Recently, a dual mode - MRI and fluorescence agent has been reported where thermodynamically stable macrocyclic Gd-DOTA chelates, and a fluorophore (dylight680) have been conjugated with a Generation 5 (G5) dendrimer (Figure 1) [25]. The pharmacokinetics of GdDOTAG5-DL680 was studied in an experimental rat model of glioma with MRI monitoring [25]. The intravenous delivery of (GdDOTA)54-G5-DL680 led to the visualization of the agent in the rat glioma tumor. In another report, a Near Infrared Red (NIR) dye, DyLight680 (DL680) was also conjugated with a dendritic PARACEST (Paramagnetic Chemical Exchange Saturation Transfer) agent to detect glioma *in vivo* in a compromised BBTB.23 Intravenous delivery of the designed nanomedicine in Figure 1 [(GdDOTA)54-G5-DL680], resulted in the agent homing into its glioma tumor site selectively. *In vivo* MRI detected the agent in a glioma tumor, but not in contralateral tissue.

The specificity of the agent was validated by whole-body NIR-optical imaging and *ex vivo* fluorescence imaging. The *in vivo* MRI showed the macroscopic location of the tumor while fluorescence imaging showed the biodistribution of the agent. A dual mode MRI-optical approach is ideally suited for *in vivo* biomedical imaging because MRI provides non-invasive *in vivo* high-resolution anatomical images, while fluorescence imaging has high sensitivity and can provide microscopic information in *ex-vivo* pathological tissues. BIRDS is a type of molecular imaging platform for magnetic resonance that utilizes the unique properties of low molecular weight paramagnetic monomers by detecting hyperfine-shifted nonexchangeable protons and transforming the chemical shift information to reflect its microenvironment (e.g., via temperature, pH, etc.). Current BIRDS studies have only used low molecular weight paramagnetic monomers (e.g., Ln-DOTP5-, Ln-DOTMA-, Ln-DOTA-4AmP5-) [36–38]. Despite their capability to accurately report molecular readouts (e.g., temperature, pH, etc.), BIRDS studies with the low molecular weight monomers suffer from short blood half-life and wide *in-vivo* distribution. To further improve the translational potential of BIRDS, we have conjugated thermodynamically stable macrocyclic p-SCN-Bn-DOTA to the amines on the surface of PAMAM dendrimers from generation 1 (G1) to generation 4 (G4) by using an isothiocyanatobenzyl group that achieves excellent synthesis yield (i.e., greater than 60%) and purities [50]. Dendrimer-based BIRDs agents demonstrated both acidic pH and temperature-sensing properties [50]. Therefore, we hypothesize that dendrimeric BIRDS agents have potential for a range of biomedical applications in quantitative theranostic imaging.

Nanodrug carriers attached to surface ligands or antibodies exploit the receptor-mediated uptake pathways that are recognized by the tumor cells [51–55]. Transferrin receptor-targeting dendrimers have demonstrated efficiency in delivering therapeutic genes and drugs to cancer cells and their use has already led to significant improvements in cancer therapies [56,57].

### Dendrimer-Based Drug Conjugate

The vasculature of GBM is fundamentally different from that of normal vasculature and offers a unique target for anti-cancer therapy. Therefore, direct targeting of tumor vasculature with vascular disrupting agents (VDAs) is distinctly different from anti-angiogenic strategies and offers a complementary approach to standard therapies. Combretastatin A4 (CA4) is a potent vascular disrupting drug but insoluble in water. CA4 was conjugated with a G3-succinamic acid PAMAM dendrimer. Conjugation of CA4 with G3 dendrimer improved water solubility as well as bioavailability [28]. However, intravenous (i.v.) delivery of G3-CA4 in an orthotopic glioma model induced necrosis at the core of the tumor leaving a rim of viable tissue. By applying the designed dendrimer-based drug conjugate strategy and tumor-specific prodrug activation mechanism, we observed the true success of inducing necrosis at the core of the tumor in an orthotopic U-251 glioma preclinical animal model [28]. Curcumin (Cur), a yellow pigment in the spice turmeric (curcuma longa), has been reported for its potential chemo preventive and chemotherapeutic activity by influencing various processes, such as cell cycle arrest, differentiation, and apoptosis in a series of cancers [58–65]. In addition, Cur inhibited proliferation, migration and invasion of GBM cells in *in-vitro* studies [66]. Nevertheless, a major criticism of Cur has been the apparent poor systemic bioavailability in *in-vivo* animal models. The latter indicates poor relevance for clinical translation even when patients are given up to 8–10grams of the free drug orally each day [63]. In addition, systemic delivery of Cur leads to non-specific distribution throughout the body [67]. It is reasonable to explore novel formulations of Cur that overcome the limitations mentioned above. A Generation 3 (G3) PAMAM dendrimer-based Cur conjugate was synthesized [27]. The synthesized G3-Cur conjugate demonstrated full solubility in aqueous media.

The *in vitro* study revealed that G3-Cur nanoparticles were internalized into glioma U-251 cells. Systemic delivery of G3-Cur conjugate led to preferential accumulation in an orthotopic preclinical glioma model and minimized systemic toxic effects. A dendrimer based G5 dual mode probe accumulated to GBM tumor and liver, respectively [25]. G3-Cur nanoparticles showed more accumulation in tumor and/or less renal uptake, likely due to small particle size, optimal surface charge, and hydrophilicity in blood [27]. Multicolor microscopy images of the tumor tissue showed that G3-Cur particles were internalized inside tumor cells selectively and further localized within nuclei. It has been demonstrated that after oral administration of 400mg of curcumin to rats only traces of unchanged drug were found in the liver and kidney. At 30 min, 90% of curcumin was found in the stomach and small intestine, but only 1% was present at 24h [68]. While Cur has shown a wide range of non-specific *in vivo* distribution, [63]G3-Cur preferentially accumulated at the tumor site. Enhanced bioavailability of G3- Cur conjugate was also observed with improved therapeutic efficacy against cells from different types of cancer. The specificity of G3-Cur was further investigated with *ex vivo* multicolor fluorescence microscopy, which showed accumulation of the particles in glioma tumor tissue selective for nuclear distribution. *Ex vivo* fluorescence microscopy showed that the G5 agent accumulated in the glioma tumor and the liver [23,25]. Therefore, conjugation of Cur to a smaller size G3 carrier improved bioavailability and tumor targeting [27].

## Conclusion

The overall ineffectiveness of small molecule chemotherapy drugs in treating malignant brain tumors can be attributed to the fact that there is only a transient elevation in drug concentrations within the extravascular extracellular compartments of tumor tissue due to the short blood half-life of small molecule chemotherapy [24]. We and others have developed dendrimer-based paramagnetic nanoparticles that cross the BBTB and accumulate in the GBM tumor site [23,69–71]. Overexpressed cell surface receptors or the process of tumor acidosis are targeted with high affinity ligands. The result demonstrates the relative accumulation of the nanomedicine to the tumor site. The delivery vehicle introduced here can be loaded with imaging agents in combination with a particular drug, thus offering the possibility of developing a nanotheranostic approach to the treatment of GBM.
